# Pigmentation in Amenorrhœa

**Published:** 1894-06

**Authors:** A. E. Aust Lawrence

**Affiliations:** Physician-Accoucheur to the Bristol General Hospital; Professor of Midwifery in University College, Bristol


### PIGMENTATION IN AMENORRHŒA.

BY

A. E. Aust Lawrence, M.D. Aberd.,

*Physician-Accoucheur to the Bristol General Hospital; Professor of Midwifery in University College, Bristol.*

Pigmentation occurs as a rule in the physiological amenorrhœa of pregancy; but it is not generally known that it often occurs in ordinary amenorrhœa, and in some cases to such an extent as to lead to the error of diagnosing the condition as Addison's disease. The case of Miss X. is one in point. She was pronounced to have this disease by two leading doctors in London; she had all the marked features of the disease, yet she has perfectly recovered, and I do not think she ever had it.

Her history briefly is: At the age of 18, after menstruating two years, she ceased to menstruate—this was in July, 1888. Discolouration of skin began in 1889, and was as well marked as a typical Addison's disease in 1890. The discolouration increased till January, 1893; then improved health was noticed, and in June, 1893, catamenia became once more established. During her ill-health she was very desponding and weak. The treatment was vinum ferri ʒj. and liquor arsenicalis ♏v. three times a day, burgundy, plenty of milk, and fresh air. She is now perfectly well and free from any discolouration.

The subject of pigmentation in connection with amenorrhœa is one of great interest, and I think that in pregnancy the absence of the catamenia has more to do with pigmentation than the presence of pregnancy; in other words, I believe the deposition of colouring matter is most likely due to the non-elimination of something that ought to be got rid of at a menstrual period, and not so much due to the altered state of the blood in pregnancy.

The whole subject of pigmentation is far too large for the present occasion, and in this note I simply wish to draw attention to its occurrence in amenorrhœa of non-pregnant women. I have noticed it in several cases, and it is important not only to enable us to avoid attributing it to a serious illness, such as Addison's disease, but also when the pigmentation is mammary to guard us from believing it to be due to pregnancy. As a rule, the pigmentation in non-pregnant amenorrhœa occurs about the face, neck, or hands; but it also does affect scars, just like one sees in pregnancy.

